# Single-Nucleotide Polymorphism Genotyping Identifies a Locally Endemic Clone of Methicillin-Resistant *Staphylococcus aureus*

**DOI:** 10.1371/journal.pone.0032698

**Published:** 2012-03-09

**Authors:** Ulrich Nübel, Andreas Nitsche, Franziska Layer, Birgit Strommenger, Wolfgang Witte

**Affiliations:** 1 Fachgebiet Nosokomiale Infektionen, Robert Koch-Institut, Wernigerode, Germany; 2 Zentrum für Biologische Sicherheit, Robert Koch-Institut, Berlin, Germany; Columbia University, United States of America

## Abstract

We developed, tested, and applied a TaqMan real-time PCR assay for interrogation of three single-nucleotide polymorphisms that differentiate a clade (termed ‘*t003-X*’) within the radiation of methicillin-resistant *Staphylococcus aureus* (MRSA) ST225. The TaqMan assay achieved 98% typeability and results were fully concordant with DNA sequencing. By applying this assay to 305 ST225 isolates from an international collection, we demonstrate that clade *t003-X* is endemic in a single acute-care hospital in Germany at least since 2006, where it has caused a substantial proportion of infections. The strain was also detected in another hospital located 16 kilometers away. Strikingly, however, clade *t003-X* was not found in 62 other hospitals throughout Germany nor among isolates from other countries, and, hence, displayed a very restricted geographical distribution. Consequently, our results show that SNP-typing may be useful to identify and track MRSA clones that are specific to individual healthcare institutions. In contrast, the spatial dissemination pattern observed here had not been resolved by other typing procedures, including multilocus sequence typing (MLST), *spa* typing, DNA macrorestriction, and multilocus variable-number tandem repeat analysis (MLVA).

## Introduction

Methicillin-resistant *Staphylococcus aureus* (MRSA) is a major cause of healthcare-associated infections [1], [2], [3]. It frequently causes outbreaks of infections that may spread within and between healthcare institutions [4]. Some strains of MRSA have demonstrably disseminated across hospitals around the globe within decades [5]. Especially at local and regional scales, however, the temporal and spatial dynamics of MRSA spread are poorly understood [6], [7]. It is usually not known, how commonly MRSA get transferred among healthcare institutions, how frequently novel strains get imported into a given medical facility, or what proportion of MRSA infections may be caused by strains that continuously circulate as endemic pathogens within a facility [8], [9]. Consequently, we currently lack important insight into the pace and range of MRSA dispersal, even though local epidemiology may have a profound impact on the cost and success of infection control measures [10], [11], [12], [13].

One reason for our limited knowledge about local-scale spread is that contemporary typing procedures identify genotypes that commonly display long-lasting persistence over entire countries or continents, and, as a consequence, provide little information about MRSA spread within a particular region [6], [7], [14], [15], [16]. For example, according to *spa* typing – a popular genotyping method based on sequence variation of a fragment from the staphylococcal *spa* gene [17] – 63% of MRSA from hospitals in all Germany currently display either one of only two types, which are dubbed *t003* and *t032*, respectively [18]. Reportedly, the frequency of individual *spa* variants is even higher in some other regions [19]. Similarly, alternative typing methods for MRSA, including DNA macrorestriction (pulsed-field gel electrophoresis) and multilocus sequence typing (MLST), result in a small number of genotypes that dominate the pathogen population over large areas [6], [7], [20], [21], [22].

Recent technological advances have made DNA sequencing faster and more cost-effective by orders of magnitude [23]. Based on genome re-sequencing data from 63 MRSA isolates affiliated with a single MLST sequence type (ST239), including 20 isolates from one hospital, a recent study suggested that genome-wide single nucleotide polymorphisms (SNPs) could potentially be exploited to discriminate related isolates within a healthcare facility [5]. However, (re-)sequencing several millions of basepairs from each MRSA isolate for routine typing in epidemiological surveillance as yet is not economically feasible. Rather, it may be useful to ascertain polymorphic sites in the genomes from a limited number of isolates in a first step, and then to subsequently apply more cost-effective means to interrogate character states of the most informative SNPs [24], [25], [26].

In a previous study, we had ascertained genome-wide SNPs through mutation discovery within an international collection of MRSA ST225 isolates [16]. Here, we have applied TaqMan PCR to track an MRSA clone that could be identified on the basis of three of those SNPs.

## Methods

### Bacterial isolates

We investigated a total of 305 MRSA isolates displaying *spa* type *t003*. Ninety-nine of these isolates were collected from hospital *X1* from 2006 through 2011, including 83 isolates from infections, nine isolates from carriers with no infections, and seven isolates with no information about clinical background. Another 16 *t003* isolates were from hospital *X2*, including five from infections, six from asymptomatic carriage, and five without infection-related information.

Hospital *X1* is a regional, general-care hospital with partial tertiary-care functions. It hosts 12 clinical departments and three interdisciplinary centers, two of which frequently exchange patients with other units. The hospital has a total of 500 beds and 50 day-care sites, and consists of a main facility and a side branch which is located 14 km apart. Hospital *X2* is another general-care hospital in vicinity to hospital *X1* (distance to *X1*, 16 km).

Further, we included 154 *t003* isolates from 51 other hospitals throughout Germany. These isolates had been sent to the German national reference center for staphylococci for genotyping and further characterization. Finally, we included 36 *t003* isolates from international sources, including the Czech Republic, Denmark, Switzerland, and the USA. The genomic diversity of these isolates had been investigated previously [16].

Upon request, we received 93 additional MRSA isolates that had been collected during the first half of 2010 from 11 hospitals located in the wider region around hospital *X1* (distance to *X1*, 30 to 148 km). Only one of these isolates turned out to have the *spa* type *t003*; it was included among the above-described strain collection for SNP typing. The proportion of MRSA among *S. aureus* isolates from hospitals in this region is 20–25%, which is similar to German average [27].

### Tree construction

We constructed a minimum spanning tree by using the Bionumerics software (Applied Maths, Ghent, Belgium). The maximum number of single-locus variants was prioritised and hypothetical nodes were allowed to decrease total tree length. Tree credibility was assessed by permutation resampling with 200 samples (see Bionumerics manual).

### *Spa* typing, DNA macrorestriction, PCR

*Spa* typing and *SmaI* DNA macrorestriction (pulsed-field gel electrophoresis) of staphylococcal isolates was performed as described previously [14]. For PCR amplification and sequencing of loci au131, au133, and au345, previously published oligonucleotide primers were used [16].

### Multilocus variable-number tandem repeat analysis (MLVA)

We investigated eight variable-number tandem repeat loci by PCR amplification and sequence analysis, applying previously described oligonucleotide primers [28]. Lengths of PCR products were determined by sequencing and used to deduce the numbers of repeats by comparison to a record of previously encountered allele sizes (provided by Leo Schouls, National Institute for Public Health and the Environment, Bilthoven, The Netherlands).

### TaqMan PCR

Using Primer 3 software (available at http://frodo.wi.mit.edu/primer3) and Primer Express 3.0 software (Applied Biosystems, Foster City, CA, USA), we designed three TaqMan SNP allelic discrimination assays. For each of the three SNPs (Table 1), a pair of amplification primers was designed, and two differentially labelled TaqMan probes to hybridize with either one of the two alleles (Table 2). TaqMan probes (Applied Biosystems, Warrington, UK) were conjugated with 3′-minor-groove binder (3′-MGB) groups [29]. Real-time PCR was performed in 10-µl reactions containing 1 µM of each primer, 80 nM of each probe, 0.2 units of Platinum *Taq* polymerase (Invitrogen, Darmstadt, Germany), 1× Platinum *Taq* PCR buffer, 1.6 mM MgCl_2_, 0.2 mM of each dNTP (Invitrogen), and approximately 15 ng of template DNA. Thermal cycling was run on a Biorad CFX96 (Biorad, München, Germany) using the following conditions: 96°C for 5 minutes and 36 cycles of 96°C for 30 seconds, 58°C for 45 seconds, and 72°C for 1 minute.

**Table 1:** Single-nucleotide polymorphisms.

SNP	Ancestral	Derived	Quality	Position in N315 genome	ORF	Annotation
ST225_au133-1	T	A	synonymous	378156	SA0321	carbohydrate kinase, PfkB family
ST225_au345-1	T	C	non-synonymous	1351084	SA1184	aconitate hydratase
ST225_au131-1	G	T	intergenic	91487	none	non-coding

**Table 2:** PCR Primers and probes.

SNP	Primers	Probes
133-1-T/A	5′-GCAATTGCAGTGGCTGTG-3′	FAM-5′-AATGATGGCATAGTCTA-3′-MGB
	5′-CCCGAATTGTTCAAATTGTTTT-3′	VIC-5′-ATGATGGCATTGTCTA-3′-MGB
345-1-T/C	5′-TGGTAAAGGTAATGACGGTGAA-3′	FAM-5′-ATTTGGCCATCAACTA-3′-MGB
	5′-TCAGGTGTTACAACACTATCAACG-3′	VIC-5′-TTTGGCCATCAATTA-3′-MGB
131-1-G/T	5′-TTCTCCGTATTGTTTCATAATAACCTC-3′	FAM-5′-CTGTAGGGGTATTATAA-3′-MGB
	5′-GATAAACACAAATGTGTCAAATACCC-3′	VIC-5′-TGTAGGGGTATTCTAA-3′-MGB

The performance of the three TaqMan assays was tested on 73 isolates with known SNP genotypes. From 11 of these isolates (chosen randomly from our collection of *t003* isolates from hospital *X1*), loci au131, au133, and au345 had been PCR-amplified and sequenced. Another 62 isolates had been screened for polymorphisms at these loci in a previous mutation discovery study [16].

## Results

### Choice of SNPs

**Figure 1:**
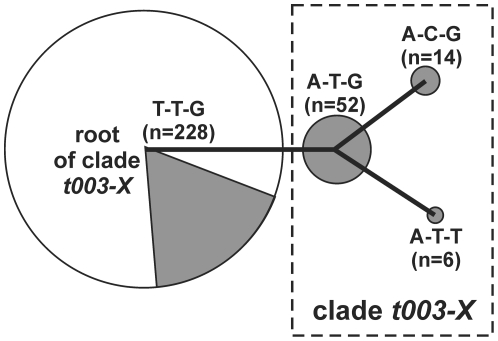
Minimum spanning tree illustrating the relationships between four SNP genotypes (corresponding to SNP allelic profiles T-T-G, A-T-G, A-C-G, A-T-T), defined through character states at three polymorphic positions in the MRSA genome (SNPs ST225_au133-1, ST225_au345-1, ST225_au131-1, respectively; see **Table 1**). The tree is fully parsimonious (i. e., there is no alternative, equally optimal solution), as each of its branches yielded 100% permutation resampling support. Circle size is proportional to the number of isolates assigned to each of the genotypes. Grey shading indicates the proportion of isolates from administrative district X, collected from 2006 through 2011. Clade *t003-X* encompasses three SNP genotypes as indicated.

From a panel of 47 SNPs that had been discovered in the course of a previously published study [16], we selected three mutations (Table 1) that define a monophyletic clade within the radiation of MRSA ST225, as illustrated in Figure 1. Character states at these bi-allelic SNPs define four different genotypes (i. e., allelic profiles, Figure 1). Notably, there were no homoplasies, as mutations at loci ST225_au345-1 and ST225_au131-1 in our present analysis were never found in the same isolate together, and they were not found in the absence of a mutation at locus ST225_au133-1. SNP ST225_au133-1 is most useful as it is phylogenetically informative and separates three genotypes (i. e., allelic profiles A-T-G, A-C-G, and A-T-T; Figure 1) from the root of the clade. These three genotypes appeared to have slightly different spatial and temporal distribution patterns. For example, only one of the genotypes was found on the neonatology and pediatrics wards (Figure S3). Further, there were extended time periods during which specific genotypes were not recovered from any infections (Figure S3). However, to avoid over-interpretation facing the small numbers of isolates assigned to each individual genotype, and for the sake of simplicity, we subsume these three genotypes under the term ‘clade *t003-X*’ in the following (Figure 1).

### Performance of TaqMan allelic discrimination assay

We used three dual-probe TaqMan 5′-nuclease PCR assays to interrogate three bi-allelic SNPs at distal positions on the MRSA genome. To facilitate single-base mismatch discrimination, TaqMan probes were equipped with 3′-MGB moieties [29]. All three assays exhibited efficient amplification of the corresponding SNP alleles (Figure S1). The minimum cycle thresholds (C_T_) varied from 12 to 19, depending on the assay and the template DNA, whereas the respective alternate alleles were not amplified (Figure S2). Of note, the observed variation of C_T_ to some extent may have been caused by the varying quantity and quality of template DNA applied, since DNA samples initially had been extracted for the purpose of *spa* typing and most of them had been stored in the freezer for several years prior to being used in the present project.

When we tested these three TaqMan assays on 73 isolates with known SNP genotypes, SNPs were called correctly for all 73 isolates. Among 305 isolates tested in total, there were four isolates that repeatedly did not yield a fluorescence signal for locus ST225_au131-1, and sequence analysis revealed that one of these isolates had a point mutation (G>T) eight basepairs upstream from the SNP, resulting in a mismatch to the TaqMan probes. In addition, there was one isolate that repeatedly did not yield amplification products for two loci, ST225_au133-1 and ST225_au345-1. Locus au345 also failed to PCR-amplify when applying previously published primers [16], suggesting this locus may have been affected by a mutation (not shown). In summary, among 915 PCR assays performed, there were six assays (0.7%) in five isolates that failed repeatedly, and 300 isolates (98%) were fully typable at all three loci. The five non-typable isolates were removed from further analyses.

### Abundance and distribution of clade *t003-X*

Among the 300 isolates fully genotyped, there were 72 isolates that displayed the derived character state at the phylogenetically informative SNP, i. e., allele A at SNP locus ST225_au133-1 (Figure 1). Among these 72 isolates, there were 14 isolates with allele C at SNP locus ST225_au345-1 (allelic profile, A-C-G) and six isolates with allele T at SNP locus ST225_au131-1 (A-T-T; Figure 1).

**Figure 2:**
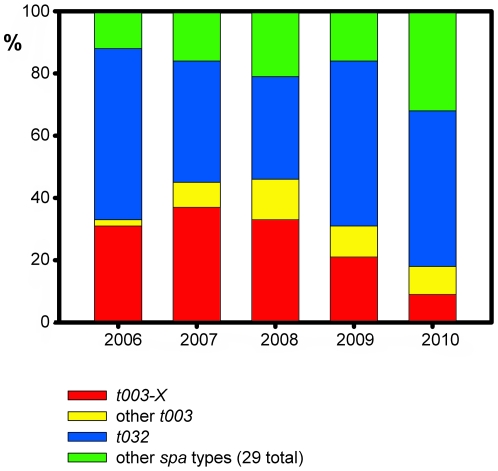
Proportion of clade *t003-X*, other *spa* type *t003* isolates, and *spa* type *t032* among MRSA from hospital *X1*. Total numbers of MRSA isolates received from hospital *X1* in individual years from 2006 through 2010 were 42, 38, 64, 60, and 74, respectively.

Remarkably, all 72 isolates in clade *t003-X* originated from only two hospitals (termed ‘*X1*’ and ‘*X2*’) within a single administrative district (termed ‘*Landkreis X*’, Figure 1). The majority (68 isolates, 94%) of *t003-X* isolates were found in hospital *X1*, where *t003-X* accounted for 9% to 33% of all MRSA that were sent for typing each year from 2006 through 2010 (Figure 2). In hospitals *X1* and *X2* together, *t003-X* accounted for 64% of *t003* isolates and was detected on ten different wards total (Figure S3). In contrast, clade *t003-X* was not found among 154 ST225/*t003* isolates from 51 other hospitals in Germany and among 36 additional isolates from Denmark, Switzerland, the Czech Republic and the USA (Figure 1). In a more targeted search, *t003-X* was also not found among 93 additional isolates from 11 hospitals located in the wider region around hospital *X1* (not shown).

### DNA macrorestriction and MLVA did not identify clade *t003-X*

A subset of *t003-X* isolates, including isolates from each year from 2006 to 2009 and representing each of the three SNP genotypes in clade *t003-X*, were analysed by *SmaI* DNA macrorestriction (pulsed field gel electrophoresis). Banding patterns from these *t003-X* isolates were identical to those from several unrelated *t003* isolates (Figure S4). Therefore, it was not possible to identify clade *t003-X* (or any of its subtypes) on the basis of DNA macrorestriction.

We genotyped a set of 26 isolates by applying a recently reported MLVA protocol (Table S1). Among all but two of the ST225 isolates, MLVA loci were almost fully conserved, and, as a consequence, MLVA was unable to identify clade *t003-X* (Table S1).

## Discussion

By applying a TaqMan real-time PCR approach, we have determined character states at three previously ascertained bi-allelic SNPs in 305 MRSA isolates affiliated to *spa* type *t003*/MLST sequence type ST225. The TaqMan PCR performed efficiently, achieving 98% typeability (the proportion of isolates that could be genotyped), and results were fully concordant with dHPLC and Sanger sequencing. Costs for reagents (including the TaqMan probes) and disposables were moderate, amounting to 0.38 € per 10-µl reaction, or 1.14 € per isolate. This price, however, is based on the assumption that several thousand isolates are to be analysed totally, such that the costly TaqMan probes will get used up completely. Moreover, it does not include the cost of labor, rent, machines etc. Miniaturization and/or multiplexing could potentially improve costs, rapidity, and throughput of real-time PCR assays [30], [31].

As a result of typing isolates from an international collection, we have identified an MRSA clade (*t003-X*) that has caused a substantial proportion of infections in a single acute-care hospital (hospital *X1*), from at least 2006 through 2010 (Figure 2). In addition, four *t003-X* isolates were detected in another hospital (*X2*) that is located only 16 kilometers away from hospital *X1*, and it is possible that this MRSA has spread between the two institutions, for example, through patient transfers. Strikingly, however, *t003-X* was not found in any other hospital out of 62 that were investigated throughout Germany nor among ST225/*t003* isolates from other countries. Consequently, *t003-X* displays a very restricted geographic distribution. It apparently has not disseminated far and its repeated introduction from outside the local area is unlikely. Rather, those point mutations that have enabled the clade's identification likely were acquired locally. In conclusion, the SNPs we have investigated enable the tracking of an MRSA clade that locally has been endemic for several years. In contrast, the spatial dissemination pattern of this MRSA had not been resolved on the basis of more convential typing procedures, including MLST, *spa* typing, DNA macrorestriction, and MLVA.

Our demonstration that a large proportion of MRSA isolates descend from a single clone calls for efforts to identify its reservoir(s), for example, through active surveillance cultures and SNP-based typing of the resulting MRSA isolates. Even though we found that *t003-X* was widespread among multiple wards within hospital *X1* (Figure S3), it remains possible that the actual reservoir is outside the hospital, for example among repeatedly re-admitted patients or in a long-term care facility serving the hospital's catchment population. Such an external source for *t003-X* could be clarified through screening of patients upon admission to the hospital.

*Spa* typing indicated that multiple different strains of MRSA occurred in hospital *X1* (Figure 2), several of which persisted from 2006 through 2010 (*t004*, *t032*, and *t105*; not shown). Notably, *spa* type *t032* was particularly prevalent (Figure 2); it is affiliated to MLST sequence type ST22, which is the most frequently found MRSA strain in Germany and several other countries in Europe [18], [19]. Our results do not exclude that these ubiquitous *spa* types conceal other clones that may be locally endemic in hospital *X1*, in addition to *t003-X*. A comprehensive investigation into the complexity of the MRSA population structure in this hospital would require a SNP discovery study covering multiple clonal complexes.

Currently ongoing and future sequencing efforts will undoubtedly lead to the discovery of abundant polymorphisms in MRSA genomes. We predict that many of these polymorphisms will be useful to identify MRSA clones that are specific to geographic regions, to individual healthcare institutions, or even beyond, for example to departments, or wards, or patient cohorts within hospitals. If future research will confirm that local endemism of specific MRSA clones is a widespread phenomenon in many healthcare institutions, informative SNPs will likely be very helpful to identify the sources of nosocomial infections in individual patients. Once suitable polymorphisms have been ascertained, cost-effective means can be employed to probe character states for tracing routes of MRSA transmission.

## Supporting Information

Figure S1 **Representative amplification curves.**
(PDF)

Figure S2 **Allelic discrimination plots.**
(PDF)

Figure S3 **Occurrence of clade** ***t003-X*** **and other** ***t003*** **isolates on ten different wards in hospital** ***X1*** **, 2006 through 2010.**
(PDF)

Figure S4 ***SmaI*** **DNA macrorestriction analysis of genomic DNA from** ***t003*** **isolates, including five** ***t003-X*** **isolates as indicated.** UPGMA clustering is based on the Dice similarity coefficient.
(PDF)

Table S1 **MLVA results.**
(PDF)
