# Supplementary figures and images for: Single-Nucleotide Polymorphism Genotyping Identifies a Locally Endemic Clone of Methicillin-Resistant Staphylococcus aureus
Source: PLoS One. 2012 Mar 9;7(3):e32698. doi: 10.1371/journal.pone.0032698 (PMC3302872; doi:10.1371/journal.pone.0032698)

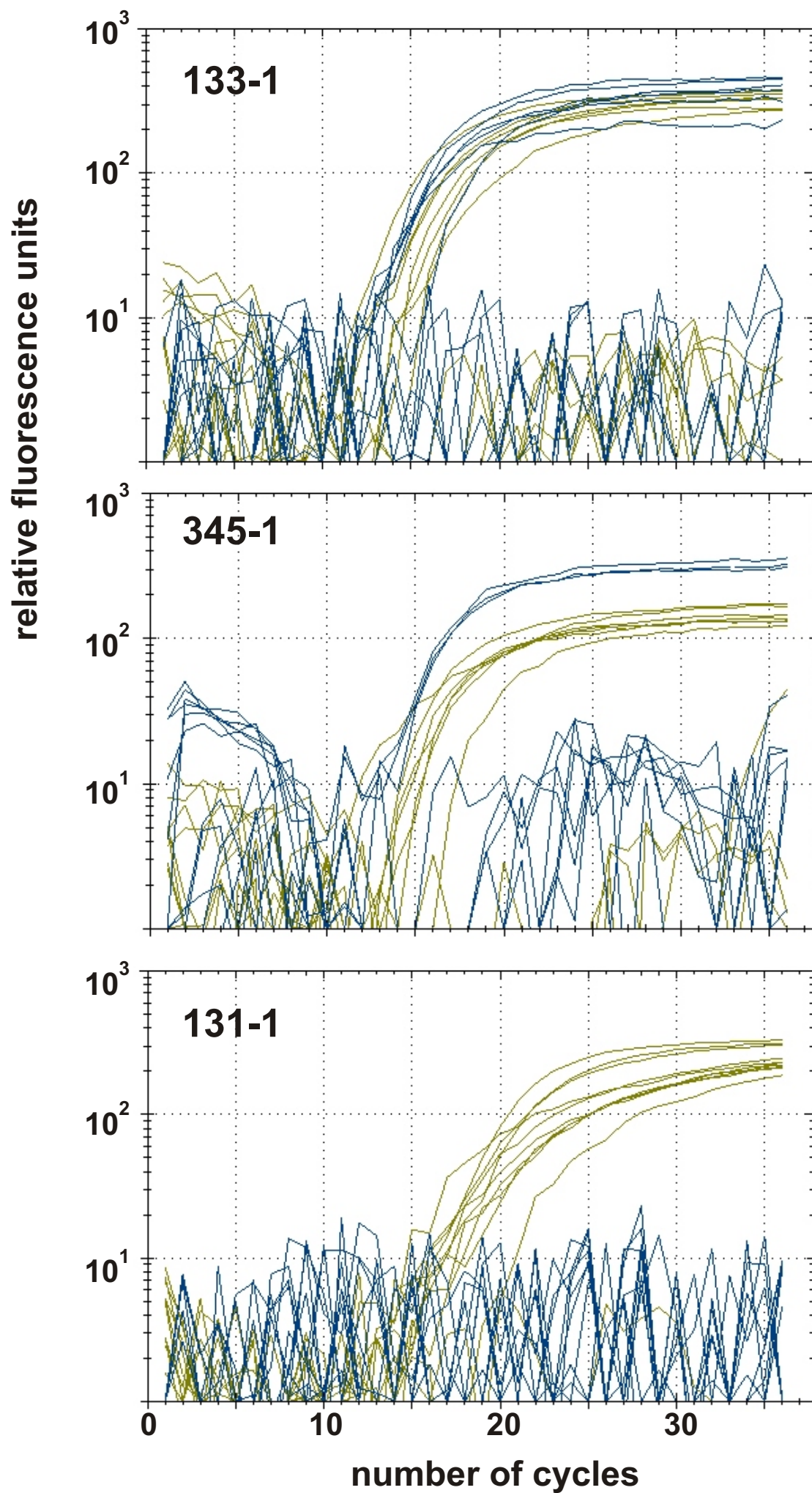

Supplement: Figure S1 — Representative amplification curves. (PDF) [file pone.0032698.s001.pdf]

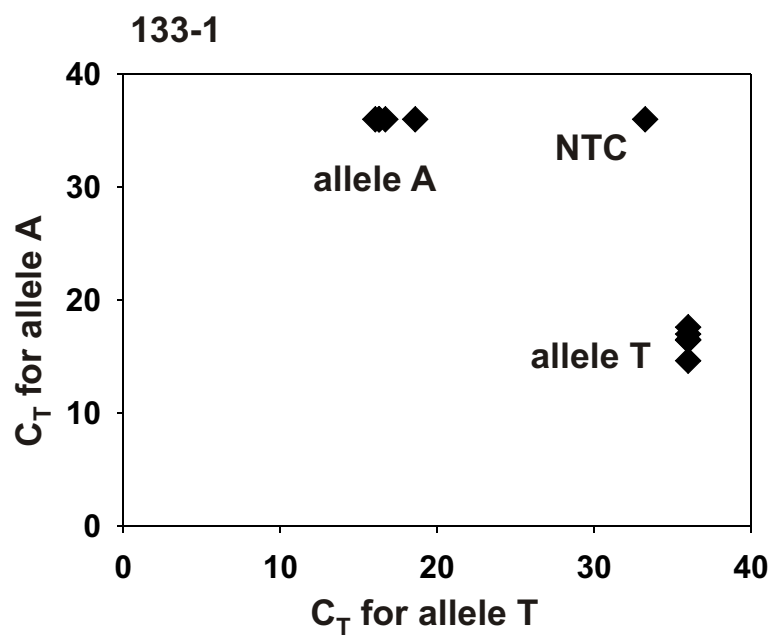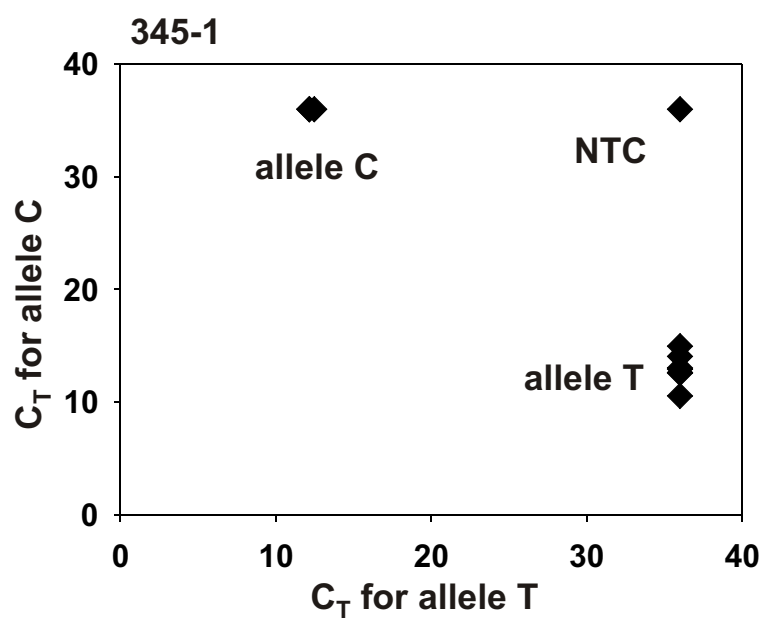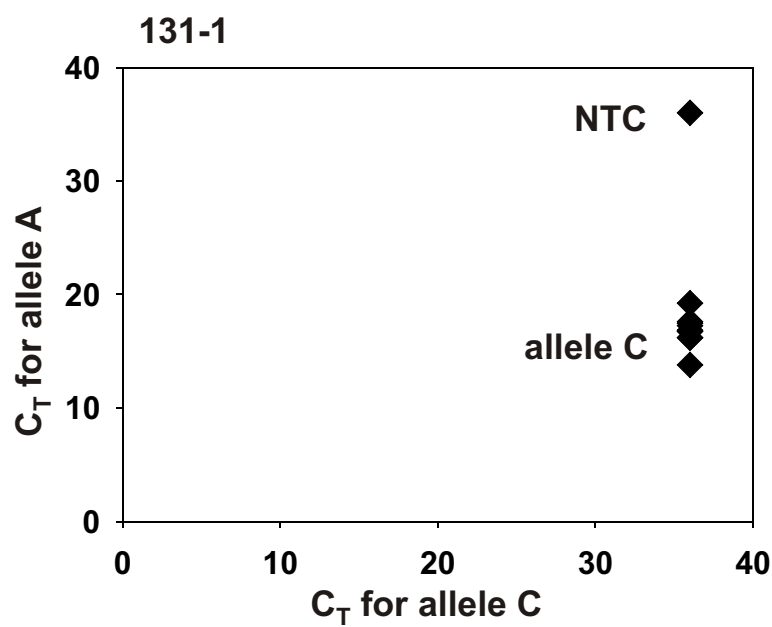

Supplement: Figure S2 — Allelic discrimination plots. (PDF) [file pone.0032698.s002.pdf]

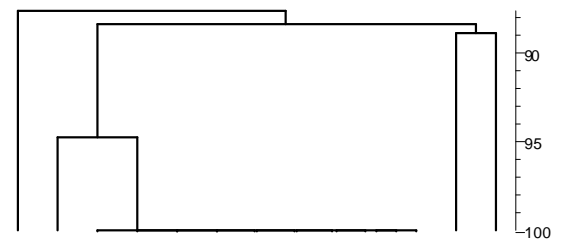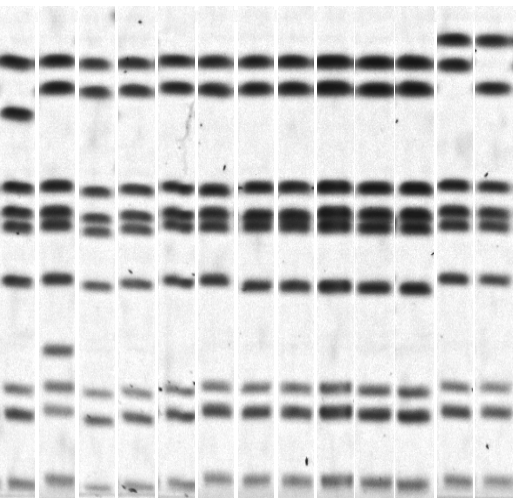

05-02010  
07-03458  
06-01602  
06-01163  
07-00265  
08-00463  
09-01692  
08-02863  
04-02981  
06-01050  
07-01367  
07-01600  
06-01100

}  
*t003-X*

Supplement: Figure S4 — SmaI DNA macrorestriction analysis of genomic DNA from t003 isolates, including five t003-X isolates as indicated. UPGMA clustering is based on the Dice similarity coefficient. (PDF) [file pone.0032698.s004.pdf]
