# Supplementary material for: Single-Nucleotide Polymorphism Genotyping Identifies a Locally Endemic Clone of Methicillin-Resistant Staphylococcus aureus
Source: PLoS One. 2012 Mar 9;7(3):e32698. doi: 10.1371/journal.pone.0032698 (PMC3302872; doi:10.1371/journal.pone.0032698)
Supplement: Table S1 — MLVA results. (PDF) [file pone.0032698.s005.pdf]

Supplementary Table. MLVA results.

Isolate	Country of origin	spa/MLST	SNP genotype	Number of repeats at each VNTR locus							
				VNTR9_01	VNTR61_01	VNTR61_02	VNTR67_01	VNTR21_01	VNTR24_01	VNTR63_01	VNTR81_01
**06-01602**	Germany	t003/ST225	t003-X	12	2	1	3	2	9	6	5
**07-00265**	Germany	t003/ST225	t003-X	12	2	1	3	2	9	6	5
**08-00463**	Germany	t003/ST225	t003-X	12	2	1	3	2	9	6	5
**06-01214**	Germany	t003/ST225	t003-X	12	2	1	3	2	9	6	5
**06-01738**	Germany	t003/ST225	t003-X	12	2	1	3	2	9	6	5
**07-00566**	Germany	t003/ST225	t003-X	12	2	1	3	2	9	6	5
**07-00911**	Germany	t003/ST225	t003-X	12	2	1	3	2	9	6	5
**06-02702**	Germany	t003/ST225	t003-X	12	99*	1	3	2	9	6	5
**06-02843**	Germany	t003/ST225	t003-X	12	2	1	3	2	99*	6	5
**08-00500**	Germany	t003/ST225	t003-X	12	2	1	3	2	99*	6	5
**08-01818**	Germany	t003/ST225	t003-X	12	2	1	3	2	9	6	5
**06-02028**	Germany	t003/ST225	t003-X	12	2	1	3	2	9	6	5
**08-02697**	Germany	t003/ST225	t003-X	12	2	1	3	2	99*	6	5
**09-01692**	Germany	t003/ST225	t003-X	12	2	1	3	2	9	6	5
**04-02981**	Germany	t003/ST225	ancestral	12	2	1	3	2	9	6	5
**09-00646**	Germany	t003/ST225	ancestral	12	2	1	3	2	9	6	5
**06-01049**	Germany	t003/ST225	ancestral	12	2	1	3	3	9	6	5
**07-00047**	Germany	t003/ST225	ancestral	12	2	1	3	2	9	6	5
**07-00320**	Germany	t003/ST225	ancestral	12	2	1	3	2	99*	6	5
**08-00013**	Germany	t003/ST225	ancestral	12	2	1	3	2	99*	6	5
**01-04209-1**	Germany	t003/ST225	ancestral	12	2	1	3	2	9	6	5
**06-01609**	Germany	t003/ST225	ancestral	12	2	1	3	2	9	6	5
**07-01598**	Czech Republic	t003/ST225	ancestral	12	2	1	3	2	99*	6	5
**07-03033**	Denmark	t003/ST225	ancestral	12	99*	1	3	2	99*	6	5
**09-00824**	USA	t002/ST225	ancestral	14	4	1	3	2	11	5	5
**N315**	Japan	t002/ST5	ancestral	14	2	1	3	2	11	6	5

99*, PCR amplification failed.
