# Supplementary material for: Single-Nucleotide Polymorphism Genotyping Identifies a Locally Endemic Clone of Methicillin-Resistant Staphylococcus aureus
Source: PLoS One. 2012 Mar 9;7(3):e32698. doi: 10.1371/journal.pone.0032698 (PMC3302872; doi:10.1371/journal.pone.0032698)
Supplement: Figure S3 — Occurrence of clade t003-X and other t003 isolates on ten different wards in hospital X1 , 2006 through 2010. (PDF) [file pone.0032698.s003.pdf]

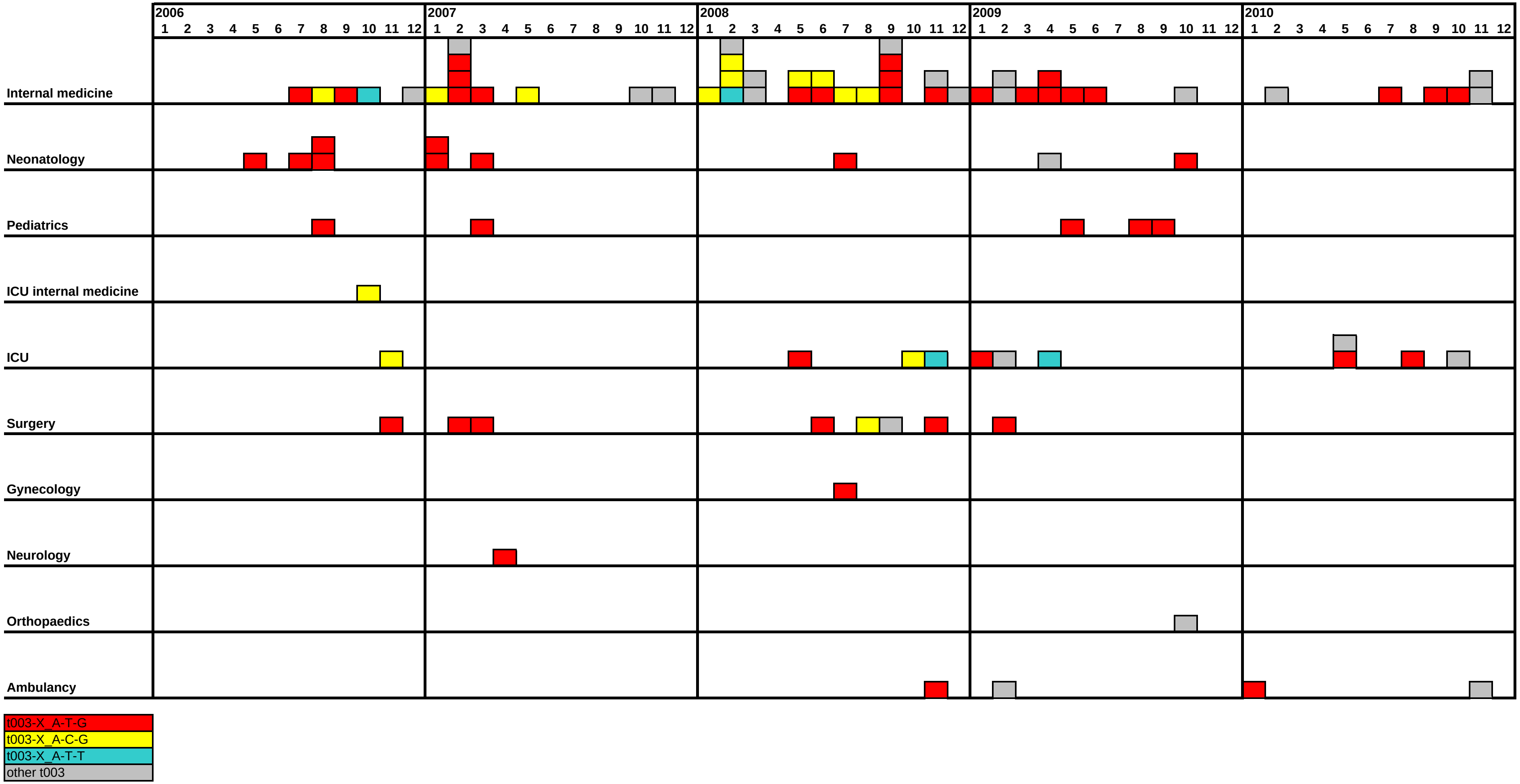

2006
2007
2008
2009
2010
1 2 3 4 5 6 7 8 9 10 11 12
Internal medicine
Neonatology
Pediatrics
ICU internal medicine
ICU
Surgery
Gynecology
Neurology
Orthopaedics
Ambulancy
t003-X_A-T-G
t003-X_A-C-G
t003-X_A-T-T
other t003
